# Demyelinating diseases of the central nervous system registry for patients with traditional Chinese medicine: Rationale and design of a prospective, multicenter, observational study

**DOI:** 10.3389/fphar.2022.981300

**Published:** 2022-11-28

**Authors:** Jia Liu, Chi Zhang, Yao Xie, Li Zhou, Li Guo, Bin Li, Zhen Jia, Jingze Zhang, Kazuo Sugimoto, Ying Gao

**Affiliations:** ^1^ Department of Neurology, Dongzhimen Hospital, Beijing University of Chinese Medicine, Beijing, China; ^2^ Institute for Brain Disorders, Beijing University of Chinese Medicine, Beijing, China; ^3^ Department of Neurology, Hunan Academy of Traditional Chinese Medicine Affiliated Hospital, Changsha, Hunan, China; ^4^ Department of Neurology, The Second Hospital of Hebei Medical University, Shijiazhuang, Hebei, China; ^5^ Neurological Laboratory of Hebei Province, Shijiazhuang, Hebei, China; ^6^ Key Laboratory of Chinese Internal Medicine of Ministry of Education and Beijing, Dongzhimen Hospital, Beijing University of Chinese Medicine, Beijing, China

**Keywords:** CNS demyelinating autoimmune diseases, multiple sclerosis, neuromyelitis optica spectrum disorders, myelin oligodendrocyte glycoprotein, Traditional Chinese Medicine, registry

## Abstract

**Background:** Traditional Chinese medicine (TCM), a main form of complementary and alternative medicine provides a potential possibility for demyelinating disease of the central nervous system (DDC) management and has been applied in considerable amounts of patients with this disorder. Nevertheless, powerful real-world evidences regarding the epidemiological and clinical characteristics, safety, and outcomes of TCM in DDC are lacking. The primary objective of the Demyelinating Diseases of the Central Nervous System Registry for Patients with Traditional Chinese Medicine (DATE-TCM) is to create an organized multicenter data collection structure to define integrative characteristics of DDC patients treated with TCM in an endeavor to fill these knowledge gaps to better inform clinical care and health policy.

**Method:** This study provides a prospective and voluntary registry by using a web-based system. Baseline data will be recorded and subsequently regular follow-up visits will be implemented every 3–6 months for a total of 5 years. The primary outcome is Annualized Aggregate Relapse Rate at 5-year follow-up.

**Results:** DATE-TCM is currently designed to capture the multidimensional (epidemiologic, demographic, clinical, etc.) features of DDC patients receiving TCM treatment, the type and long-term safety and efficacy of TCM intervenes in the DDC populations, as well as the interaction of TCM treatments and disease modifying therapies in the management of DDC, aiming to include 2000 eligible adult DDC patients with TCM intervenes from 35 participating centers, covering 77.4% of provincial administrative regions of mainland China.

**Conclusion:** DATE-TCM is the first, largest, most geographically extensive, and standard registry-based observational study that systematically document the real-world data regarding the TCM application in the DDC populations, which will be extraordinarily important for clarifying the comprehensive characteristics and outcomes of TCM in DDC, further shed light on standardizing and optimizing the TCM measures for DDC management and establishing evidence-based clinical practice guidelines for TCM application in DDC.

**Clinical Trial Registration:** clinicaltrials.gov, identifier NCT05415579.

## 1 Introduction

Demyelinating Diseases of the Central Nervous System (DDC) is a collective term for a group of autoimmune-mediated inflammatory disorders in young adult age, mainly including multiple sclerosis (MS), Neuromyelitis optica spectrum disorder (NMOSD), and myelin oligodendrocyte glycoprotein (MOG)-associated disease (MOGAD), characterized by myelin loss and axonal damage of the central nervous system (CNS) (Chen et al., 2021). Depending on the functional neuronal system affected, there is a diversified spectrum of possible symptoms of DDC, such as paresthesia, limb weakness, visual impairment, balance disturbance, and bladder or bowel dysfunction, resulting in neurological disability of the patients (Hoftberger and Lassmann, 2017). MS is thought to occur most frequently in Western Europe and North America, while the highest incidence of NMOSD has been reported in Afro-Caribbean region (Bishop and Rumrill, 2015; Papp et al., 2021). In China, epidemiological data suggests that the incidence of NMOSD (0.278/100,000 person years) is slightly higher than that of MS (0.235/100,000 person years) (Tian et al., 2020a; Tian et al., 2020b). Since MOGAD is a newly defined disease entity in 2018, its epidemiological information within specific regions or worldwide has not yet been clarified (Lopez-Chiriboga et al., 2018). Currently, a range of injectable disease-modifying therapies (DMTs) such as glatiramer acetate, several interferon-beta regimens, natalizumab, rituximab, satralizumab, and oral DMTs such as fingolimod, dimethyl fumarate, teriflunomide have become available for DDC treatment (McGinley et al., 2021; Wallach et al., 2021). However, not all patients respond well to their prescribed DMTs, manifesting limited efficacy and side effects (progressive multifocal leukoencephalopathy, infections, gastrointestinal side-effects, leukopenia, arthralgia) that may require additional management.

Patients suffering from DDC are more likely to seek help from complementary and alternative medicine (CAM), which is used by 57%–81% of patients with MS in developed country (Nayak et al., 2003; Apel et al., 2006; Schwarz et al., 2008; Ng and Kishimoto, 2021). Traditional Chinese medicine (TCM), a major form of CAM, including Chinese herbal medicine (CHM), acupuncture and other non-medication therapies provides a potentially valuable possibility for DDC treatment. A prospective clinical study investigating factors associated with CAM usage in MS patients in the United States indicated that the three most common CAMs used by patients with MS were chiropractor, massage, and acupuncture, and the most frequent reasons for CAM use were symptom relief, back problems, and pain (Kim et al., 2018). A systematic review and meta-analysis including 20 randomized-controlled trials (RCTs) and 1,100 participants assessing the efficacy and safety of CHM as an adjunct therapy of patients with MS demonstrated significant effects of CHM on improving Expanded Disability Status Score (EDSS), annual relapse frequency, and overall clinical efficacy rate compared with conventional western treatments (Song et al., 2017). Also, in this review, six studies monitoring the adverse effects of CHMs in MS treating showed that CHMs were well tolerated in all patients. In addition, acupuncture has been reported to be effective in reducing spasticity, pain, and numbness and improving fatigue, imbalance, and motor function in patients with DDC (Criado et al., 2017; Khodaie et al., 2022). Nevertheless, it is worth noting that poor methodological quality, comparatively prominent clinical heterogeneity, and small sample size are common problems in most RCTs on TCM. Therefore, favorable evidence-based studies remain scarce and many physicians remain skeptical about the use of TCM in DDC management, which directly affects the feasibility of TCM treatment and limits the widespread application of TCM in the DDC populations.

Although RCTs provide useful information on the effectiveness of DDC therapies, lines of evidence suggests that long-term and prospective databases or registries of large inception cohorts could include various domains such as demographic, disease course, medication status, socio-economic data, and auxiliary examination results, which is expected to provide the best understanding of the natural history, epidemiology, and clinical characteristics of DDC and the long-term effectiveness of treatments that cannot be captured in any other way. Accordingly, regional or national MS or DDC registries have been established for decades, including the German MS registry, the Italian MS registry, the Danish MS registry, the Swedish MS registry, the Norwegian MS Registry and Biobank, the European Database for MS, the North American Research Committee on MS (NARCOMS), and the MSbase, which support comparative effectiveness studies, provide important insights into the epidemiology and outcomes of DDC, and offer valuable patient perspectives. (Flachenecker and Stuke, 2008; Flachenecker et al., 2014; Traboulsee, 2014). In China, there are several single- or multi-centers prospective registries for DDC (NCT03514030, NCT04101058, NCT04106830, NCT05154370, ChiCTR2000030651), all of which are based in Western medicine hospitals and mainly focus on elucidating the epidemiologic, clinical, imaging features of DDC, as well as the treatment response to DMTs. To the best of our knowledge, there is still a lack of powerful real-world evidence regarding the epidemiological and clinical characteristics, safety, and outcomes of TCM in DDC. Therefore, the primary objective of the Demyelinating Diseases of the Central Nervous System Registry for Patients with Traditional Chinese Medicine (DATE-TCM) is to define the multidimensional (epidemiologic, demographic and clinical, etc.) characteristics of DDC patients receiving TCM treatment, the type and long-term safety and efficacy of TCM intervenes in DDC populations, and the interaction of TCM treatments and DMTs in the management of DDC, with the aim of filling the above knowledge gaps and provide a better inform for clinical care and health policy.

## 2 Method and analysis

### 2.1 Study design

DATE-TCM is a prospective, multi-center, observational, disease-based registry of DDC patients treated with TCM in a real-world setting, designed to determine the general characteristics of DDC patients treated with TCM, the types of TCM intervenes, the long-term safety and efficacy of TCM treatment, and the interaction of TCM intervenes and DMTs in the management of DDC. The registry protocol follows the Declaration of Helsinki and Tokyo guidelines for humans and was approved by the Ethics committee of Dongzhimen Hospital, Beijing University of Chinese Medicine (2022DZMEC-171-02) as the coordinating center and will be approved by the local ethics committee of all participant centers. Prior to inclusion in the DATE-TCM, patients or their legally authorized representatives are informed of their conduct by medical staff and must sign a written informed consensus before data collection commences. In this consent form, patients can give permission for relevant biological specimens to be collected and their data to be used for further research activities. Patient participation will be entirely voluntary, with the ability to withdrawal from DATE-TCM participation at any time, and without the need to provide an explanation.

### 2.2 Participants

#### 2.2.1 Key inclusion criteria

(1) Male or female participants aged ≥18 years old;
(2) Diagnosis of DDC according to relevant criteria or consensus, including MS, NMOSD, and MOGAD (Wingerchuk et al., 2015; Lopez-Chiriboga et al., 2018; Thompson et al., 2018);
(3) Patients are treated with TCM intervenes, including the CHM, acupuncture, moxibustion, massage, taiji, and qigong;
(4) Obtain informed written consent from the patient, and/or his/her legally authorized representatives.

#### 2.2.2 Key exclusion criteria

(1) Refusal to give informed consent;
(2) Malignancies, infectious diseases (HBV, HCV, HIV, etc.), congenital or acquired severe immunodeficiency, significant cardiovascular, pulmonary, and hepatic diseases or conditions;
(3) Mental disturbance or severe cognitive impairment impeding necessary information collection and assessment.

### 2.3 Site selection and study sample size

**FIGURE 1:**
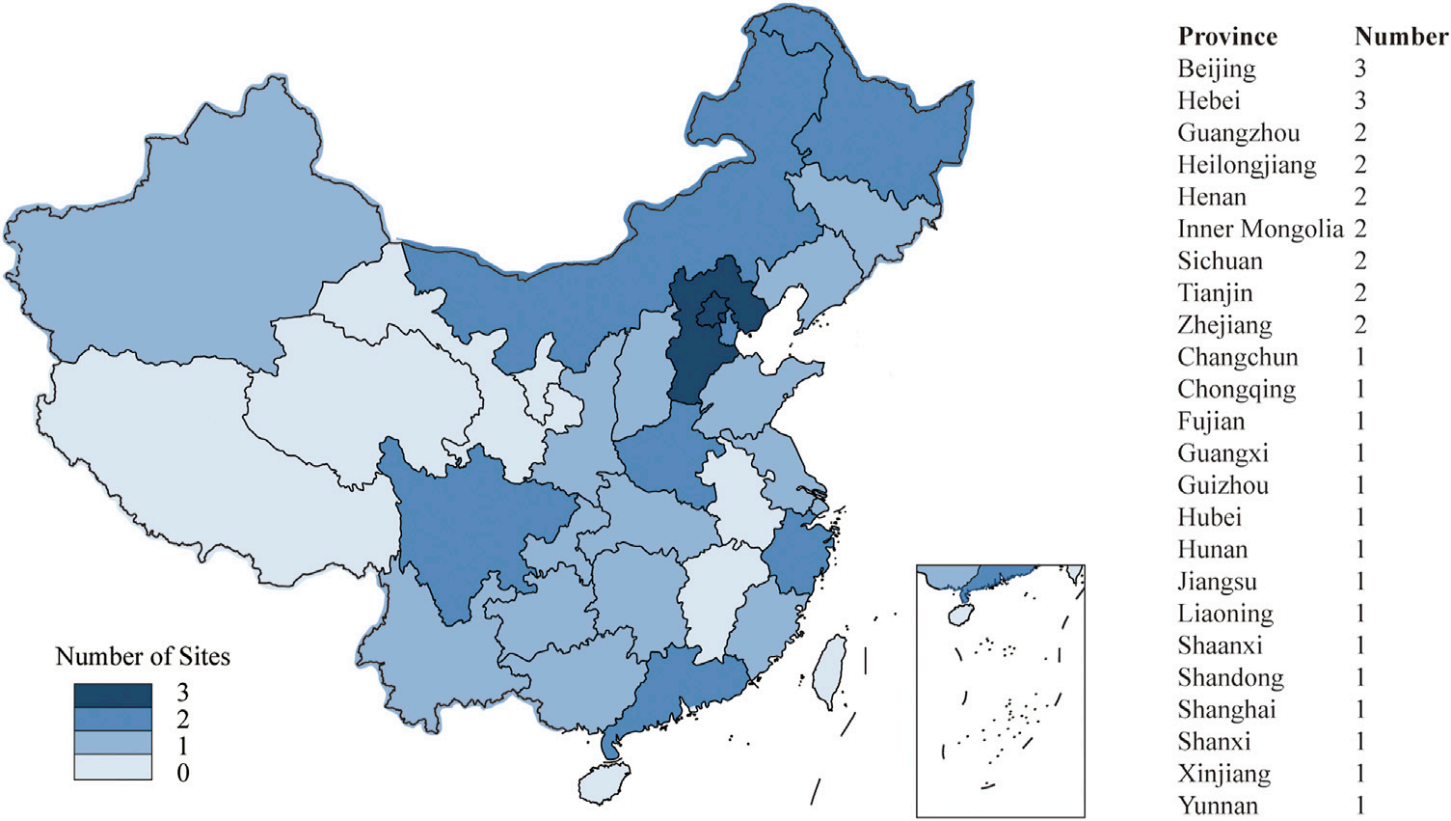
Sites distribution of DATE-TCM.

Participant centers were recruited nationwide from each region of mainland China on a voluntary basis. They must meet the following criteria: 1) the ability to accurately diagnose DDC; 2) the ability to admit over 30 patients with DDC per year; 3) be able to complete relevant data upload and biospecimens collection in accordance with the database requirements. Currently, DATE-TCM possesses a total of 35 qualified participant centers, covering 77.4% of provincial administrative regions in mainland China (Figure 1). And the number of participating centers is gradually expanding, with the goal of exceeding 50. Based on estimate of at least 30 cases treated per participating center annually, the total number of patients for the 35 centers is 1,050 per year. By the end of the second year after enrollment commences, up to 2000 patients are expected to be enrolled. DATE-TCM is expected to begin patient recruitment on 20 June 2022.

### 2.4 Data collection and follow-up plan

The main variables were determined with reference to the MS or DDC Registry in Germany, Italy, Denmark, Sweden, Norway, Switzerland, as well as the European Database for MS, NARCOMS, and MSbase (Butzkueven et al., 2006; Hillert and Stawiarz, 2015; Myhr et al., 2015; Trojano et al., 2019; Magyari et al., 2021; Marrie et al., 2021; Ohle et al., 2021), and combined with the characteristics of TCM treatment (Table 1). To be noted, the compulsory common minimum dataset (MDS) was setup by expert consensus and reference to other regional or national MS or DDC registry studies, consisting of information selected to ensure that 1) sufficient data were collected to define multidimensional characteristics of the DDC populations receiving TCM treatment; 2) data collection was simple enough; 3) each participant center could achieve maximum completeness and quality; 4) facilitate the exchange and collaboration with national and international datasets. DATE-TCM database only accepts records with the completed MDS (Supplementary Table S1). In addition to the collection of data information, DATE-TCM involves the collection and storage of blood specimens for subsequent testing according to an established schedule.

**TABLE 1:** Main variables and follow-up plan in the DATE-TCM.

Index	Variables	Baseline visit	Follow-up visit Every 3 months ◎
			Every 6 months ☉
			Every 12 months •
Demographic variables	Gender, year of birth, ethnicity, birthplace, present residence	○	
Socioeconomic variables	Education, working situation, occupational or educational changes due to DDC, societal context (e.g. medical insurance), marriage, reproduction, walking assistance, Full-time Nursing, TCM-related expense, Total disease-related expense	○	◎
Diagnosis variables	First symptoms and lesion locations, year of onset, diagnosis, year of diagnosis, diagnostic criteria, symptoms (lesion locations) ever, attack list (date, lesion locations or symptoms)	○	
Symptoms variables	Symptoms current, clinical type of MS, time to SPMS	○	◎
Comorbidities variables	Comorbidities, medication for comorbidities	○	◎
Risk factors and medical history	Smoking consumption, Alcohol consumption, Family history, Allergy history, Trauma history, Vaccination, Sun exposure	○	
Treatment	Any previous medication (start and end date), reasons for discontinuation	○	
	DMT current (Generic name, date of start, dose, frequency), TCM type current (TCM preparation, acupuncture, moxibustion, massage, taiji, qigong), detailed use of TCM preparations (name of TCM preparation or information on commercial products, date of start, dose, frequency, composition of TCM preparation including herbal, animal, mineral, and other natural materials, forms of TCM preparation including pills, powders, soft extracts, pellets, decoctions, and medicated liquor), Additional medicine/supplements	○	◎
Safety assessments	AEs, SAEs		◎
CSF	LP date, LP performed at (relapse/remission), Number of Mono and leukocytes, protein content, OCB, IgG index, anti-AQP4 IgG, anti-MOG IgG	○	◎
MRI	MRI date, T2 lesions (total number), New or enlarged T2 lesions (number), Gd-enhanced T1 lesions (total number), Quantitative/volumetric MRI, Brain Parenchymal fraction, Normalized total brain volume, Normalized total lesion volume in white matter	○	•
Other examinations	Evoked potentials (Visual, Somatosensory, Brainstem)	○	◎
	Blood leukocyte count and classification count, Blood liver and renal functions test, Serum NFL, Serum cytokines, Serum anti-AQP4 IgG, Serum anti-MOG IgG	○	☉
Specific scales	BMI, EDSS	○	◎
	MSFC, SDMT, FSS, MSIS, EQ-5D	○	☉
Four examinations of TCM	Inspection, listening and smelling examination, inquiry, palpation	○	◎
Relapse	Relapse date, Affected functional system, severity, Any treatment performed, recovery after relapse		◎

Note: DDC, demyelinating Diseases of the Central Nervous System; MS, multiple sclerosis; SPMS, secondary progressive multiple sclerosis; DMT, disease modifying therapy; TCM, traditional Chinese medicine; CSF, Cerebro-Spinal Fluid; AEs, adverse events; SAEs, severe adverse events; LP, lumbar puncture; OCB, oligoclonal bands; AQP4, Aquaporin-4; MOG, myelin oligodendrocyte glycoprotein; MRI, magnetic resonance imaging; NFL, neurofilament light chain; BMI, body mass index; EDSS, expanded disability status scale; MSFC, multiple sclerosis functional composite; SDMT, symbol digit modalities test; FSS, fatigue severity scale; MSIS, multiple sclerosis impact scale; EQ-5D, EuroQol-5D.

The follow-up plan was developed with reference to the relevant elements of DDC registry studies from other regions or countries and the characteristics of TCM treatment. Demographics, symptoms, and comorbidities variables, treatment status, safety assessments, Cerebro-Spinal Fluid (CSF) or other examinations (e.g., evoked potentials) results, Body mass index (BMI), EDSS, four examinations of TCM, and relapse information will be follow-up every 3 months by telephone or face-to-face visits. The Multiple Sclerosis Functional Composite (MSFC), Symbol Digit Modalities Test (SDMT), fatigue severity scale (FSS), Multiple Sclerosis impact scale (MSIS), and EuroQol-5D (EQ-5D) assessments will be administrated every 6 months through face-to-face visits. Blood leukocyte count and classification count, blood liver and renal functions test, serum neurofilament light chain (NFL), serum cytokines, serum anti-AQP4 IgG, and serum anti-MOG IgG will be tested every 6 months. MRI related evaluation of brain and spinal cord will be conducted every 12 months.

### 2.5 Treatment

DATE-TCM does not restrict the application of western medical treatment methods, and related information will be systematically recorded. Patients are commonly treated with high dose of intravenous steroid during the relapse phase, which usually refers to intravenous administration of 1 g or 500mg of glucocorticoid per day for 3-5 consecutive days, halved every 3 days. Plasma exchange and immunoabsorption therapies are also optional treatment in the relapse phase. Immunomodulatory therapies including low dose steroid, immunosuppressants (Azathioprine, Mycophenolate, etc.), and DMTs (fingolimod, Teriflunomide, Rituximab, satralizumab, etc.) are necessary for the remission phase. In addition, DATE-TCM will particularly detailed document the application of TCM, such as the timing of TCM application (relapse or remission phase), the types of TCM (defined according to the World Health Organization including the CHM, acupuncture, moxibustion, massage, taiji, qigong, etc.), and CHM relevant information (dose, frequency, duration, and adherence to TCM treatment of herbs, herbal materials, herbal preparations, or finished herbal products) in the entry and follow-up visits (Song et al., 2022).

### 2.6 Outcome measurements

The primary and secondary outcomes are listed in Table 2. The primary outcome is the Annualized Aggregate Relapse Rate, defined as the number of confirmed relapses within a year. A relapse is defined as the appearance of a new or worsening previously stable or improving pre-existing neurological abnormality, separated by at least 30 days from onset of a preceding relapse. Such abnormalities must be present for at least 24 h and occur in the absence of fever or infection.

**TABLE 2:** The outcomes of DATE-TCM study.

**Primary outcomes**
Annualized Aggregate Relapse Rate
**Secondary outcomes**
Total number of adverse events during evaluation
Percentage of participants with adverse events
Time to 3-month sustained disability progression
Time to 6-month sustained disability progression
Number of new or newly enlarging T2 hyperintense lesions as measured by magnetic resonance imaging (MRI)
Number of gadolinium-enhancing T1-weighted lesions as measured by MRI
Percent change in brain volume as measured by MRI
Change in multiple sclerosis functional composite (MSFC) score
Change in symbol digit modalities Test (SDMT) score
Chance in fatigue severity scale (FSS) score
Change in euroQol- 5 dimension (EQ-5D) score
Change in multiple sclerosis impact scale (MSIS) score

The Secondary outcomes include total number of adverse events (AE, any unfavorable and unintended sign, symptom, syndrome, or illness observed by the investigator or reported by the participant during the study), percentage of participants with AE, time to 3-month sustained disability progression (disability progression was defined as an increase from baseline in EDSS score of at least 1-point or at least 0.5-point for participants with baseline EDSS score >5.5), time to 6-month sustained disability progression, number of new or newly enlarging T2 hyperintense lesions, number of gadolinium-enhancing T1-weighted lesions, percent change in brain volume, change in MSFC score, change in SDMT score, chance in FSS score, change in EQ-5D score, and change in MSIS score.

### 2.7 Platform and quality control

Authorized and qualified medical staffs at each center could input data into a web-based electronic data capture (EDC) system designed by Institute for Brain Disorders, Beijing University of Chinese Medicine (IBD-BUCM) with a unique ID *via* computers or mobile devices. To avoid duplicate patient entries, the patients’ identifying data (name, gender, date of birth, national identification number) is entered into the registry at the time of enrolment and is used to further generate a unique pseudonym. This online data collection platform also allows and facilitates the implementation of additional data modules required by specific studies or new research questions. Although DATE-TCM currently only has data collection templates in Chinese, data capture modules in Japanese and English will be added in near future to allow and encourage more regional and national centers to participate in this registry.

Good data quality is critical to the utility of the registry and the validity of the conclusions drawn from the data. Prior to registry initiation, participating centers are provided with training materials and tutorials. The entered datasets are checked for duplicates, completeness, plausibility, and consistency. Those with missing or inconsistence values are restricted to uploading or returned for correction. In order to continuously ensure high data quality, the DATE-TCM has established extensive data quality control measures: 1) a standardized EDC system for data collection with a uniformed template for each participant center, and the main variables and follow-up plan in the template was determined with reference to the relevant content from established DDC registries in other regions or countries and the characteristics of TCM treatment; 2) branching logic (based on previous responses and subsequently asking only relevant questions) to ensure that data is entered only into applicable fields; 3) cross-reference-checks to compare data between different questions and forms; 4) optimal completeness is achieved by independent case confirmation methods using multiple sources of notification, and validity is ensured by application of diagnostic criteria method; 5) additional check will be carried out by related specialists of IBD-BUCM every 3 months, and the validated data will be locked by the system for later analysis.

DATE-TCM is highly concerned with data security. Measures are in place to ensure data security, including email confirmation during the account creation process, password strength monitoring, and strict separation of personally identifiable information from research data.

### 2.8 Research activities

DATE-TCM is a scientific research registry and data collected through the registry should be used for research and healthcare issues with a high priority and not for other purposes. The Scientific Steering Committee of DATE-TCM, including clinicians, methodologists, representatives of participant centers will evaluate research proposals based on the scientific quality, feasibility, and value of the project. Once the Scientific Committee has completed its evaluation of the applications, a contract will be drawn up for each project, setting out the rules regarding data property and budget. The researchers involved will then be allowed access to a specific range of data.

### 2.9 Data analysis

As for the continuous variable, normality test was performed for all index awaiting assessment. If it is in accordance with normal distribution, it will be described as mean with 95% confidence interval (CI) or with standard deviation (SD), and one-way analysis of variance (ANOVA) was used for comparison in 3 or more groups, independent sample *t*-test was used for comparison in 2 groups, and Pearson correlation was used for correlation analysis between index. If not, it will be described as median with range or interquartile range (IQR), and Kruskal–Wallis one-way ANOVA was used for comparison among 3 or more groups, Mann-Whitney *U* test was used for comparison between 2 groups, and Spearman correlation was used for correlation analysis between index. Regarding categorical variable, it will be presented as frequencies or proportions, and tested by Fisher exact test. Kaplan-Meier method consists of calculating the probabilities of non-occurrence of event at any observed time of event. The Cox proportional-hazards regression model will be used to investigate the association between the survival time or time of event of patients and one or more predictor variables. Subjects with incomplete data will also be included in the analysis. For each variable, the number of subjects with missing data will be documented. Defining the multidimensional characteristics of DDC patients receiving TCM treatment, the types and detailed information of TCM in DDC treatment, as well as possible combinations of TCM treatments and Western medications are significant objectives of DATE-TCM. Based on these objectives, we did not impose any restriction on patients receiving TCM intervenes or western medications or both at different centers, and the relevant information was recorded systematically for further relevant descriptive studies. Notably, determining the long-term safety and efficacy of TCM interventions in the DDC population and the interaction of TCM treatments with DMTs are important goals for the establishment of DATE-TCM as well. The related studies could be performed based on the established registry data with subsequently systematic cases screening and identification criteria for different study purposes (e.g., the efficacy of a specific TCM preparation or a specific combination of TCM preparation and DMT) in order to obtain a relatively consistent treatment context of patients in a given study. Moreover, propensity score methods will be applied in these comparative studies to control for confounding factors to ensure baseline comparability between groups before evaluating the impact of a treatment on expected clinical end point events. Additionally, we will continue to appropriately adapt the detailed statistical methods according to the characteristics and needs of each DATE-TCM-based study. Values of *p* < 0.05 were considered to indicated statistical significance. All data sets were analyzed using SPSS 21.0 Software or Program R (Version 3.5.1).

## 3 Discussion

Globally, a couple of regional or national patient registry studies have been performed, providing valuable information with respect to the disease characteristics of DDC. Currently, there are two large prospective cohort studies on DDC in China, namely the China National Registry of CNS Inflammatory Demyelinating Diseases (CNRIDD, NCT05154370) and the Clinical and Imaging Patterns of Neuroinflammation Diseases in China (CLUE, NCT04106830), with study start dates of 15 December 2021 (estimated) and 1 January 2019 (actual), respectively. The CNRIDD, CLUE and DATE-TCM share some similarities and differences: 1) Number of patients: The CNRIDD, CLUE, and DATE-TCM are expected to included 10,000 (child and adult), 1,000 (adult), and 2000 (adult) patients, respectively; 2) Follow-up schedule: The follow-up period of CNRIDD, CLUE and DATE-TCM are 5, 1, and 5 years, respectively; 3) Study objective: Similar to other international registry studies, the CNRIDD and CLUE were dominated by the clinicians or researchers of western medicine and were mainly dedicated to elucidating the epidemiology, clinical, imaging, and prognostic features of DDC itself, as well as the safety and efficacy of western medications, especially DMTs (Hurwitz, 2011; Flachenecker, 2014). In these studies, there was no specific mention of whether and what information to collect regarding the application of TCM in the DDC populations. With the addition of the MSIS, EQ-5D and FSS scales, the DATE-TCM is the first and largest clinician- and patient-driven study designed to detailed elucidate the comprehensive characteristics and outcomes of TCM in DDC, which is expected to contribute to complementing the general characteristics of Chinese and Asian DDC populations and optimizing guidelines for complementary alternative medicine, particularly herbal medicine, in the management of DDC.

In addition to the systematic documentation of TCM application data in DDC, the DATE-TCM also delivers the following advantages: 1) A distinguishing feature of the Chinese national healthcare system is that TCM and western medicine complement and cooperate with each other to guarantee the overall health of the Chinese people. Correspondingly, the choice between conventional and TCM approaches in DDC treatment is not necessarily either/or in China. Thus, DATE-TCM do not impose additional restrictions on the type of participating centers, which allows our study to include not only Chinese medicine hospitals, but also Western medicine hospitals, as well as a certain number of neurological or specialized rehabilitation hospitals or centers. The above facilitates the inclusion of a larger DDC populations in the database. 2) The evaluation of the severity and significance of specific symptoms and their consequences for disease management often varies between physicians and patients. Subsequently, patients-reported outcomes (PROs) are becoming increasingly important for disease monitoring. Based on these, the DATE-TCM not only includes the clinician-reported medical records such as diagnosis, disability status, relapses, and use of DMT, but also has several PROs modules by application of specific scales involving FIS, EQ-5D, and MSIS to better define fatigue, quality of life, disease burden, and disease impact in DDC populations. 3) Validated blood biomarker assays. DATE-TCM includes not only the collection of data information, but also the collection of peripheral blood samples and the detection of related biomarkers such as NFL (a biomarker of neuronal damage), anti-MOG antibody, and anti-AQP4 antibody (biomarkers for diagnosis, disease activity and treatment responsiveness monitoring). These contribute to an objective and multidimensional monitoring and evaluation of the use of TCM in DDC. 4) A significant number of DDC patients are being or have been treated with TCM in regions and countries other than China. In order to better cover a wider range of TCM users, data collection templates in Japanese and English are being constructed. In addition, primary variables and MDS were determined with reference to MS or DDC Registry studies in multiple regions and counties, which allows our data entries to be relatively consistent with other registry studies and facilitates subsequent collaborative and comparative studies to reveal the general characteristics of TCM in DDC management in different geographical areas and the interaction between TCM and western medicine, especially DMT drugs, and standardize and optimize the overall DDC treatment schemes. 5) DATE-TCM provides a relatively long-term follow-up platform (expected to be at least 5 years) for DDC patients, which helps to interpret the long-term safety and efficacy of TCM in DDC and the long-term impact of TCM on the disease course of DDC patients.

DATE-TCM still has certain biases or limitations. Firstly, the DATE-TCM is a voluntary registration study and there is no legal obligation to report DDC cases in China at present, which makes our registry non-population based. Secondly, the existing legal barriers make inclusion of under-age DDC patients quite challenging, and DATE-TCM currently only includes adult DDC patients. Thirdly, we have certain inclusion criteria for participating centers, making it impossible for some local lower-level providers (e.g., community health centers) to be included, which may result in a potential loss in the number of patients.

In conclusion, DATE-TCM is the first, largest, most geographically extensive, and standard registry-based observational study that systematically document the real-world data regarding the TCM application in the DDC populations, which will be extraordinarily important for clarifying the comprehensive characteristics and outcomes of TCM in DDC, further shed light on standardizing and optimizing the TCM measures for DDC management and establishing evidence-based clinical practice guidelines for TCM application in DDC.

## Data Availability

Data are available on reasonable request.
