# Supplementary material for: Demyelinating diseases of the central nervous system registry for patients with traditional Chinese medicine: Rationale and design of a prospective, multicenter, observational study
Source: Front Pharmacol. 2022 Nov 28;13:981300. doi: 10.3389/fphar.2022.981300 (PMC9744113; doi:10.3389/fphar.2022.981300)
Supplement: Supplementary file 1 [file Table1.docx]

| **Supplemental Table 1. Minimum datasets of DATE-TCM** | |
| --- | --- |
| Demographic variables | Gender, Year of birth, Birthplace, Present Residence |
| Socioeconomic variables | Education, Working situation, Marriage, Reproduction, TCM-related expense |
| Diagnosis variables | First symptoms and lesion location, year of onset, year of diagnosis, diagnosis, diagnostic criteria, symptoms (lesion locations) ever, attack list (date, lesion locations or symptoms) |
| Symptoms variables | Symptoms current, clinical type of MS |
| Comorbidities variables | Comorbidities |
| Treatment | Any previous medication (start and end date), reasons for discontinuation |
|  | DMT current (Generic name, date of start), TCM type current (TCM preparation, acupuncture, moxibustion, massage, taiji, qigong), detailed use of TCM preparations (name of TCM preparation or information on commercial products, date of start, dose, frequency, composition of TCM preparation including herbal, animal, mineral, and other natural materials, forms of TCM preparation including pills, powders, soft extracts, pellets, decoctions, and medicated liquor) |
| Safety assessments | AEs, SAEs |
| CSF | LP date, LP performed at (relapse/remission), OCB, IgG index, anti-AQP4 IgG, anti-MOG IgG |
| MRI | MRI date, T2 lesions (total number), New or enlarged T2 lesions (number), Gd-enhanced T1 lesions (total number) |
| Specific scales | BMI, EDSS |
|  | MSFC, SDMT, FSS, MSIS, EQ-5D |
| Relapse | Relapse date, Affected functional system, Any treatment performed |
| Other examinations | Blood leukocyte count and classification count, Blood liver and renal functions test, Serum NFL, Serum anti-AQP4 IgG, Serum anti-MOG IgG |

**Note**: MS, multiple sclerosis; DMT, disease modifying therapy; TCM, Traditional Chinese medicine; CSF, Cerebro-Spinal Fluid; AEs, adverse events; SAEs, severe adverse events; LP, Lumbar puncture; OCB, oligoclonal bands; AQP4, Aquaporin-4; MOG, Myelin oligodendrocyte glycoprotein; MRI, Magnetic Resonance Imaging; BMI, Body mass index; EDSS, Expanded Disability Status Scale; MSFC, Multiple Sclerosis Functional Composite; SDMT, Symbol Digit Modalities Test; FSS, fatigue severity scale; MSIS, Multiple Sclerosis impact scale; EQ-5D, EuroQol-5D.
